# Sex-Specific Associations of Gut Microbiota Composition with Sarcopenia Defined by the Asian Working Group for Sarcopenia 2019 Consensus in Older Outpatients: Prospective Cross-Sectional Study in Japan

**DOI:** 10.3390/nu17101746

**Published:** 2025-05-21

**Authors:** Daisuke Asaoka, Kazuya Toda, Shin Yoshimoto, Noriko Katsumata, Toshitaka Odamaki, Noriyuki Iwabuchi, Miyuki Tanaka, Jin-Zhong Xiao, Yuriko Nishikawa, Osamu Nomura, Tsutomu Takeda, Akihito Nagahara, Shigeo Koido, Toshifumi Ohkusa, Nobuhiro Sato

**Affiliations:** 1 Department of Gastroenterology, Juntendo Tokyo Koto Geriatric Medical Center, Tokyo 136-0075, Japan; onomura@juntendo.ac.jp; 2 Department of Microbiota Research, Juntendo University Graduate School of Medicine, 2-1-1 Hongo, Bunkyo-ku, Tokyo 113-8421, Japan; shin-yoshimoto923@morinagamilk.co.jp (S.Y.); n_katumt@morinagamilk.co.jp (N.K.); t-odamak@morinagamilk.co.jp (T.O.); n-iwabuchi@morinagamilk.co.jp (N.I.); j_xiao@morinagamilk.co.jp (J.-Z.X.); ynishika@juntendo.ac.jp (Y.N.); ohkusa@juntendo.ac.jp (T.O.); nsato@juntendo.ac.jp (N.S.); 3 Innovative Research Institute, Morinaga Milk Industry Co., Ltd., Zama 252-8583, Kanagawa, Japan; kazuya-toda983@morinagamilk.co.jp (K.T.); m_tanaka@morinagamilk.co.jp (M.T.); 4 Department of Gastroenterology, Juntendo University School of Medicine, Tokyo 113-8421, Japan; t-takeda@juntendo.ac.jp (T.T.); nagahara@juntendo.ac.jp (A.N.); 5 Division of Gastroenterology and Hepatology, Department of Internal Medicine, The Jikei University Kashiwa Hospital, 163-1 Kashiwa-shita, Kashiwa 277-0004, Chiba, Japan; shigeo_koido@hotmail.com

**Keywords:** gut microbiota, sarcopenia, elderly, Japan

## Abstract

**Background/Objectives:** Sarcopenia (SA), an age-related impairment in skeletal muscle mass and function, is related to gut microbiota (GM) through inflammation and short-chain fatty acid (SCFA) generation. However, data on this relationship in older Japanese adults remain limited. We investigated the relationship of GM composition with SA, based on the Asian Working Group for Sarcopenia (AWGS) 2019 criteria, among elderly Japanese outpatients. **Methods:** Between June 2022 and January 2023, this prospective cross-sectional study enrolled 356 community-dwelling outpatients aged ≥ 65 years at the Department of Gastroenterology, Juntendo Tokyo Koto Geriatric Medical Center. SA was determined based on the AWGS 2019 consensus criteria. GM was analyzed using 16S rRNA gene sequencing, and alpha/beta diversity, taxonomic composition, detection rates, and correlations with skeletal muscle mass index (SMI), grip strength, and gait speed were investigated. **Results:** Among 356 (144 males, 212 females) participants, 50 (35 males, 15 females) had SA. Differences in GM diversity and composition were primarily noted among male participants. Men with SA had lower alpha diversity and distinct beta diversity profiles. Six bacterial genera—*Eubacterium I*, *Fusicatenibacter*, *Holdemanella*, *Unclassified Lachnospira*, *Enterococcus H*, and *Bariatricus*—had lower abundances in the SA group. Several of these genera showed positive correlations with SMI, grip strength, and gait speed. Conversely, no differences in GM characteristics were seen among females. **Conclusions:** GM composition was associated with SA among older Japanese men. These sex-specific differences emerged consistently, highlighting the potential of microbiota-based strategies for SA prevention in older males.

## 1. Introduction

Sarcopenia (SA) involves age-related impairment in skeletal muscle mass (MM), muscle strength (MS), and physical function. SA is not merely a musculoskeletal disorder; it is a critical contributor to frailty, disability, and loss of independence in the elderly population. It leads to reduced mobility, diminished autonomy, and an increased risk of institutionalization and mortality. Frail individuals with SA face significantly higher risks of falls, hospitalization, functional decline, and death. The loss of muscle strength and function is closely linked to difficulties in performing activities of daily living (ADLs) and instrumental activities of daily living (IADLs) [1]. Early detection and targeted interventions are essential to mitigate its impact and preserve quality of life in older adults. Epidemiological studies estimate that 10–16% of older adults worldwide are affected by SA. However, prevalence varies depending on the population studied and the diagnostic criteria used [2]. In Japan, the Asian Working Group for Sarcopenia (AWGS) 2019 Consensus criteria is widely utilized to determine risk among individuals [3]. Risk factors for SA include physical inactivity, malnutrition, smoking, extreme sleep duration, and metabolic disorders. In terms of pathophysiology, it has been reported that age-associated imbalance between anabolic and catabolic processes on the protein synthesis pathway in skeletal muscles, a decrease in the size and number of type II muscle fibers, fat infiltration into muscles, and a decrease in the number of satellite cells [4]. The condition is associated with higher risks of falls, fractures, cognitive impairment, and mortality, as well as longer hospital stays and postoperative complications. SA interferes with walking and daily activities, predisposes to falls, and reduces social engagement. It is a major contributor to prolonged care dependence in older individuals. While SA can be mitigated through appropriate exercise and nutritional interventions, optimal nutritional strategies remain to be fully elucidated.

Recent advances in metagenomic technologies have provided further information about gut microbiota (GM) and its systemic effects. The gut-muscle axis is known as a crucial modulator of age-related muscle health, acting through pathways involving inflammation, immune regulation, energy metabolism, endocrine signaling, and insulin sensitivity [5]. GM may participate in the occurrence of SA by modulating the butyrate and bile acid synthesis metabolism, inducing chronic low-titer inflammatory reaction, promoting insulin resistance, regulating muscle synthesis metabolism, affecting the host immune system, damaging tight junctions of intestinal epithelial cells, and enhancing intestinal permeability through various pathways [6,7]. Glycine betaine, tryptophan, bile acids, and butyric acid are the most promising biomarkers among putative mediators [8,9,10]. In particular, it has been reported that GM changes with age in the elderly, and it is possible that this may affect muscle function through gut metabolites. Dysbiosis of GM can cause heightened intestinal permeability, i.e., “leaky gut,” promoting systemic inflammation via translocation of bacterial toxins and pro-inflammatory molecules. This chronic low-grade inflammation leads to SA progression [11]. Notably, SA frequently coexists with chronic disorders including diabetes, cardiovascular disease, and obesity [12,13,14]. GM can influence SA by regulating inflammation and synthesizing short-chain fatty acids (SCFAs), such as butyrate, propionate, and acetate [15,16]. These acids are key microbial metabolites that have anti-inflammatory effects and influence host energy metabolism. Consequently, improving the intestinal environment is an innovative technique for disease prevention, with probiotics and prebiotics being considered effective interventions for maintaining health.

The GM of the Japanese population is known to be strongly shaped by distinct dietary habits and genetic factors, resulting in a microbial composition that differs markedly from that of other populations. For instance, traditional Japanese dietary patterns have been shown to significantly influence the diversity and structure of the intestinal microbiota [17], with beneficial bacteria such as *Bifidobacterium* and *Blautia* commonly dominating [18]. In addition, many Japanese individuals possess genetic polymorphisms (SNPs) associated with reduced lactase activity, which may facilitate the colonization and proliferation of *Bifidobacterium* [19]. Given these unique dietary and genetic influences, the Japanese GM exhibits a distinctive profile. Therefore, it is of particular importance to investigate the relationship between GM and SA specifically within the Japanese population. However, despite the growing interest in this topic, the exact mechanisms underlying the association between GM and SA remain poorly understood, especially in older Japanese populations. We explored relationships of GM composition with SA among older outpatients attending a university-affiliated geriatric medical center, based on the AWGS 2019 diagnostic consensus.

## 2. Materials and Methods

### 2.1. Study Design

Between June 2022 and January 2023, this prospective cross-sectional study used fecal samples from patients aged ≥ 65 years who were outpatients at the Department of Gastroenterology, Juntendo Tokyo Koto Geriatric Medical Center. We performed 16S rRNA-based GM analysis [20] using DNA extracted from feces and compared the alpha diversity, beta diversity, abundance, and detection rates of bacterial genera between SA and non-SA patients. Additionally, we investigated the correlations between bacterial genera and various MS indicators, including indices of MM, MS, and gait speed.

We included individuals aged 65–89 years who were assessed for SA within the past year according to the AWGS 2019 consensus criteria [3] and provided written informed consent. We excluded individuals with (i) neurological disorders such as Parkinson’s disease, Huntington’s disease, normal pressure hydrocephalus, progressive supranuclear palsy, epilepsy, multiple sclerosis, cerebral infections, or complications from head trauma; prior major depression or bipolar disorder; alcohol or other substance abuse; multiple infarctions, brain tumors, or subdural hematomas; or cognitive impairment due to vitamin B12 or folate deficiency, or neurosyphilis; (ii) severe diseases (e.g., advanced cardiovascular, hepatic, renal, gastrointestinal, or endocrine-metabolic disorders) or active gastrointestinal malignancy or inflammatory bowel disease; (iii) use of health supplements or foods containing probiotics, oligosaccharides, or dietary fiber; (iv) severely abnormal blood pressure or blood tests, severe anemia, drug or food allergies, heavy smoking or drinking, or an irregular lifestyle (e.g., inconsistent meal patterns or sleep duration); (v) use of anti-dementia drugs, psychoactive drugs, or severe diabetes mellitus requiring insulin treatment; and (vi) individuals deemed ineligible by the principal investigator.

### 2.2. Definition of SA and MS Indicators

SA was diagnosed based on the AWGS 2019 Consensus criteria [3]. Handgrip strength was assessed two times in both hands using a handgrip dynamometer (Toei Light Co., Ltd., Saitama, Japan), with the larger value recorded as the maximum MS. MS < 28 kg for male patients and <18 kg for female patients was considered to be reduced, in accordance with the AWGS criteria. Gait speed was manually assessed using a stopwatch and defined as slow when <1.0 m/s, as per the AWGS criteria. Lean mass and regional fat were assessed using whole-body DXA (Prodigy Advance, GE Healthcare, Tokyo, Japan), according to the standard protocol. Whole-body fat and lean mass were recorded for arms, legs, and trunk. Appendicular lean mass was calculated as the sum of the lean mass of the upper and lower limbs, and the appendicular skeletal muscle mass index (SMI) was recorded by dividing the appendicular lean mass by height squared. A low appendicular skeletal MM was considered to be SMI < 7.0 kg/m^2^ in male patients and <5.4 kg/m^2^ in female patients.

### 2.3. Fecal DNA Preparation and Microbiota Analysis

Fecal DNA was prepared and analyzed for microbiota as previously described with slight modifications [20]. Briefly, fecal samples were collected using the Techno Suruga stool collection kit (brush type). DNA was extracted from fecal samples, and purified DNA was suspended in 2000 µL of Tris-EDTA buffer (pH 8.0). PCR amplification and DNA sequencing of the V3–V4 region of the bacterial 16S rRNA gene were performed on the Illumina MiSeq instrument (Illumina, San Diego, CA, USA). The obtained paired-end FASTQ data from the registered data in the cohort study were trimmed and merged before selection of the amplicon sequence variants (ASVs). The classification and diversity analysis of the ASVs were performed using the QIIME2 software package, version 2022.8 (https://qiime2.org/ accessed on 29 December 2022), as described previously [9]. Taxonomic classification was performed using the naive Bayes classifier trained on Greengenes2, version 2022.10 (https://greengenes2.ucsd.edu/ accessed on 2 February 2023), with a 99% threshold for full-length sequence operational taxonomic units. After the assignment of each ASV to a bacterial species, alpha diversities were calculated using QIIME2 software. Furthermore, principal coordinate analysis based on Bray–Curtis dissimilarity was performed using R software (ver. 4.3.1) using the vegan (version 2.6-4) and ape (version 5.7-1) packages.

### 2.4. Statistical Analysis

We assessed 398 individuals for inclusion. After informed consent, 42 individuals who refused to undergo fecal sampling were excluded, and 356 completed the final examination and were included in FAS (Full Analysis Set). Quantitative variables are presented as mean ± standard deviation. Statistical analyses were performed using R software and EZR (ver. 4.2.2) [21]. The Mann–Whitney U test was used to analyze background information, alpha-diversity, and genera of bacteria. Fisher’s exact test was used to analyze the detection rate of bacteria genera. Permutational analysis of variance for principal coordinate analysis was conducted in R using the adonis2 function from the vegan package, version 2.6-4. Spearman’s rank correlation coefficient was employed to determine associations between gut bacterial genera and muscle-related variables.

## 3. Results

### 3.1. Baseline Characteristics

**Figure 1:**
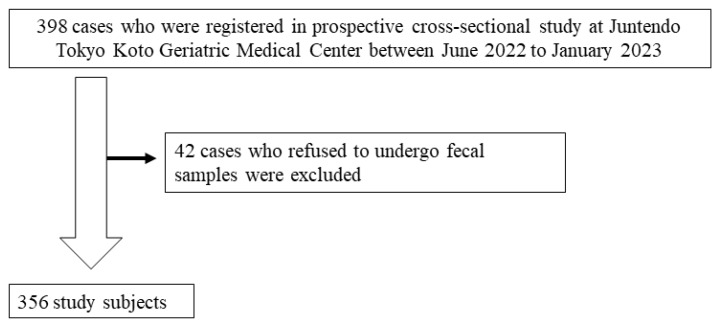
Flow chart of study participants.

Figure 1 presents a flow chart of study participants. We assessed 398 individuals, of whom 356 met the eligibility criteria. Among these, 50 had SA, whereas 306 did not (Table 1). Of the 144 male participants, 35 had SA and 109 did not. Of the 212 females, 15 had SA and 197 did not. Males with SA were significantly older than those without SA. Both male and female SA patients had lower body mass index, SMI, grip strength, and gait speed compared to non-SA patients.

A total of 398 outpatients were enrolled, and 356 patients with gut microbiota data were investigated.

### 3.2. GM Analysis Between SA and Non-SA Individuals

**Figure 2:**
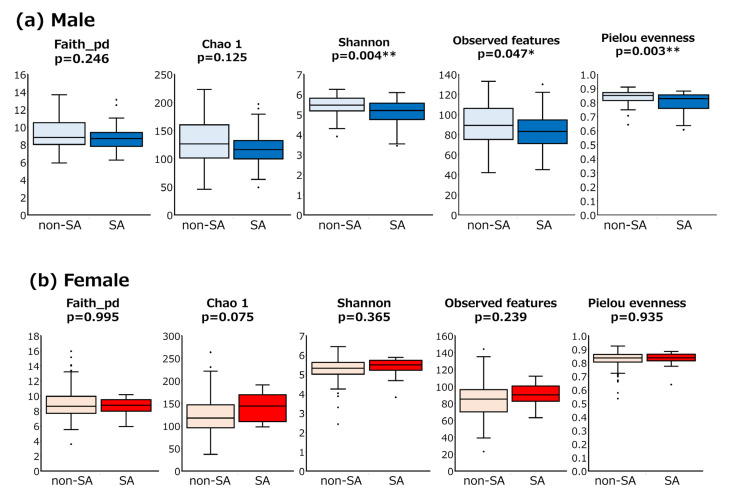
**Gut microbiota α-diversity analysis between sarcopenic and non-sarcopenic participants.** Comparative analysis of α-diversity between sarcopenic and non-sarcopenic participants in male (**a**) and female (**b**). α-diversity (Faith_pd, Chao 1, Shannon, observed features and Pielou evenness indices) was measured of male SA (*n* = 35) and non-SA (*n* = 109) and female SA (*n* = 15) and non-SA (*n* = 197) participants. Box plots show the median, as well as the lower and upper quartiles. Whiskers represent the minimum and maximum spread. Statistical significance of differences was assessed using the Mann–Whitney U test (* *p* < 0.05, ** *p* < 0.01).

**Figure 3:**
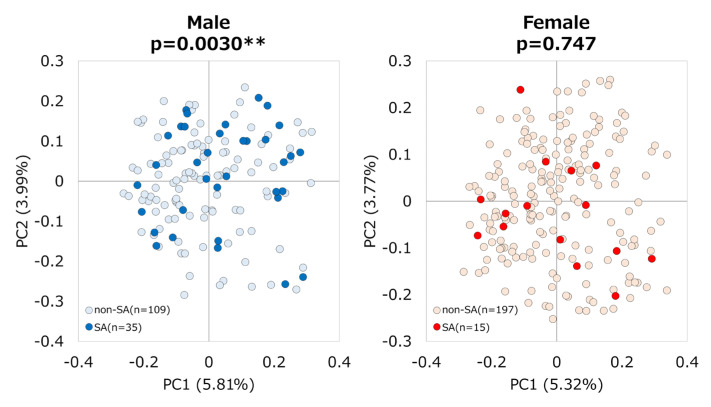
**PCoA based on Bray–Curtis analysis between sarcopenic and non-sarcopenic participants.** PCoA plots show the beta-diversity with Bray–Curtis dissimilarity. Each dot represents an individual participant. The statistical significance of differences in beta-diversity was analyzed using PERMANOVA (** *p* < 0.01).

β-diversity was significantly different between male and female participants (*p* = 0.0001) (Supplementary Figure S1). Therefore, subgroup analyses were conducted by sex. In male SA (*n* = 35) and non-SA (*n* = 109) groups, some indexes of α-diversity (Shannon (*p* = 0.004), observed features (*p* = 0.047), and Pielou evenness (*p* = 0.003)) were lower in the SA group (Figure 2a). Furthermore, the β-diversity of GM was significantly different between the two groups (*p* = 0.0030) (Figure 3). In contrast, for female subjects, no significant differences in either alpha or beta diversity were found between the two groups (Figure 2b and Figure 3).

### 3.3. Abundance of Bacterial Genera Between SA and Non-SA Patients

**Figure 4:**
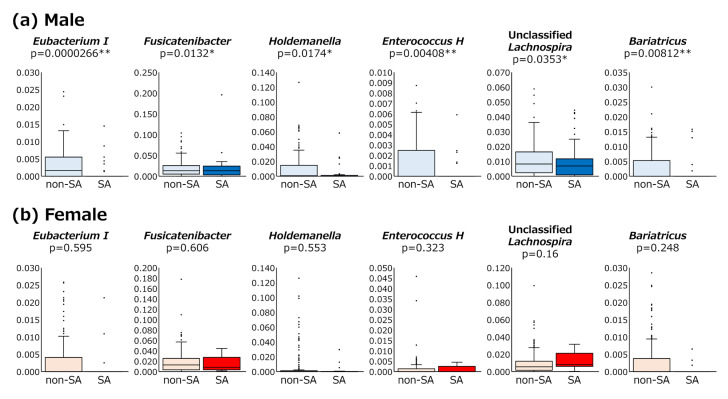
**Abundance of bacterial genera between sarcopenic and non-sarcopenic patients.** Significant differences in genera present in the gut microbiota between sarcopenic and non-sarcopenic participants in males (**a**) and females (**b**). Schematic box plots show the median of relative abundance, as well as the lower and upper quartiles. Whiskers represent the minimum and maximum spread. Statistical significance of differences was assessed using the Mann–Whitney U test (* *p* < 0.05, ** *p* < 0.01).

At the genus level, the relative abundances of six bacterial genera, *Eubacterium I* (*p* = 0.000026), *Fusicatenibacter* (*p* = 0.0132), *Holdemanella* (*p* = 0.0174), *Enterococcus H* (*p* = 0.00408), *Bariatricus* (*p* = 0.0353), and *Unclassified Lachnospira* (*p* = 0.00812), were lower in the male SA group (Figure 4). However, no significant differences were observed between female SA and non-SA groups.

### 3.4. Detection Rates of Bacterial Genera in SA and Non-SA Patients

Regarding the detection rates of the six bacterial genera, four of the six genera (*Eubacterium I* (*p* = 0.0002), *Holdemanella* (*p* = 0.009), *Enterococcus H* (*p* = 0.002), and *Bariatricus* (*p* = 0.01)) had decreased detection rates in the male SA group. Detection rates were not significantly different in the female group (Table 2).

### 3.5. Correlations Between Bacterial Genera and MM and MS

**Figure 5:**
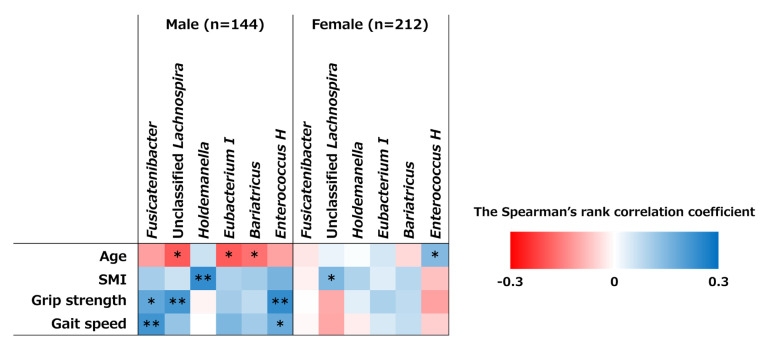
Correlations between bacterial genera and indices of muscle mass and strength. A heat map illustrated the Spearman rank correlation coefficients between the 6 bacterial genera relative abundance and age, skeletal muscle index (SMI), grip strength, and gait speed (* *p* < 0.05, ** *p* < 0.01).

Spearman correlation analysis revealed significant positive correlations between *Fusicatenibacter* and *Enterococcus H* and grip strength and gait speed in male participants. Unclassified *Lachnospira* was positively correlated with grip strength, and *Holdemanella* was positively correlated with SMI. However, in females, no significant correlation was observed between these genera and grip strength or gait speed (Figure 5).

## 4. Discussion

In this prospective cross-sectional study, we assessed the association between GM composition and SA, as defined by the AWGS 2019 consensus [3], in elderly outpatients attending a university hospital clinic specializing in geriatric care. This is the first study in Japan to investigate this association in a clinical elderly population. The association between SA and GM composition differed by sex. Specifically, significant differences in GM diversity and abundance between SA and non-SA individuals were observed only in male participants, whereas such differences were not evident in females. These findings indicate a sex-specific pattern in the relationship between GM and SA, rather than sex differences in SA or GM individually.

Among male participants, the SA group exhibited lower alpha diversity compared to the non-SA group. Furthermore, the beta diversity also differed significantly between the two groups. At the genus level, six bacterial genera—*Eubacterium I*, *Fusicatenibacter*, *Holdemanella*, *Unclassified Lachnospira*, *Enterococcus H*, and *Bariatricus*—were significantly reduced in abundance in the SA group. Of these, four genera also showed lower detection rates in the male SA group. Several of these genera produce SCFAs, particularly butyrate, and may be involved in maintaining skeletal MM and function [15,16]. Conversely, no significant differences in GM diversity or composition were noted between female SA and non-SA groups. This sex disparity aligns with previous studies. For instance, Park et al. demonstrated that SMI was linked to greater alpha diversity in males, while no such difference was found in females in a large sample of middle-aged individuals [22,23]. Other studies have similarly noted sex-specific patterns in GM composition and functional pathways [24]. Testosterone production declines with age, and testosterone treatment has been suggested to have beneficial effects on muscle mass and function. The association between testosterone decline and GM involves complex interactions, particularly through microbial enzymes such as β-glucuronidase and sulfatase. A study has identified several gut bacteria with significant β-glucuronidase activity, including species from the genera *Clostridium*, *Bacteroides*, and *Escherichia*. These bacteria contribute to the modulation of androgen levels by regulating the balance between conjugated and free testosterone in the gut [25]. Studies have shown that microbial sulfatases are widespread among gut bacteria, particularly within the phyla *Bacteroidetes* and *Firmicutes* [26]. A decline in beneficial microbial populations or enzyme activity may lead to reduced reactivation of testosterone, contributing to lower systemic levels and associated clinical manifestations. Aging is associated with decreased activation of key signaling pathways involved in muscle growth, such as the mTOR pathway, leading to decreased muscle protein synthesis [27]. These observations suggest that sex hormones, age-related endocrine changes, or other physiological differences may underlie the observed sex differences, although further research using larger cohorts is warranted to confirm these findings.

The reduction in SCFA-producing genera in SA individuals observed in this study supports earlier reports that linked lower SCFA levels with diminished muscle function [11,15,28]. The observed reduction in SCFA-producing genera among SA individuals in this study is consistent with several prior investigations reporting GM alterations in SA. For example, Kang et al. [29] reported that butyrate-producing genera such as *Lachnospira*, *Fusicatenibacter*, *Roseburia*, *Eubacterium*, and *Lachnoclostridium* were significantly less abundant in individuals with SA, while *Lactobacillus* was more prevalent. Similarly, Ticinesi et al. [10] found that SA individuals had different fecal microbial profiles, with reduced abundances of SCFA-producing species such as *Faecalibacterium prausnitzii* and *Roseburia inulinivorans*, as well as *Alistipes shahii*. Lou et al. [30] reported a negative correlation between the abundance of *Escherichia-Shigella* and relative SMI. Furthermore, *Lachnospira* was positively correlated with skeletal MM. Zhang et al. [31] found that SA individuals had lower abundances of common gut bacteria such as *Bacteroides*, *Faecalibacterium*, *Fusobacterium*, and *Prevotella*, and higher abundances of pathogens such as *Escherichia-Shigella* and *Klebsiella*. They also reported impaired amino acid biosynthesis and protein processing pathways in the SA group, suggesting possible disruption of protein metabolism. Moreover, Zhou et al. [32] identified specific genera such as *Blautia*, *Lachnospiraceae_unclassified*, and *Subdoligranulum* as potential microbial biomarkers for SA.

These findings are further supported by a recent systematic review, which reported that multiple SCFA-producing genera—including *Lachnospira*, *Fusicatenibacter*, *Roseburia*, *Eubacterium*, *Lachnoclostridium*, and *Slackia*—were consistently reduced in individuals with age-related SA [33]. This reinforces the hypothesis that dysbiosis of SCFA-producing bacteria may play a critical role in SA pathophysiology through impaired microbial contributions to host energy and protein metabolism. In men, a reduction in short-chain fatty acid (SCFA)-producing bacteria may be associated with SA, consistent with findings reported in other countries. In contrast, in women, distinct pathogenic mechanisms may be involved, potentially arising from the combined effects of hormonal factors and the unique GM composition and dietary habits characteristic of the Japanese population.

Additionally, studies focusing on the Japanese population have also linked microbial profiles with muscle health. Sugimura et al. [34] observed that increases in *Blautia* and *Eggerthella* and decreases in *Faecalibacterium* were related to MS in older Japanese adults. Sugimura et al. [35] also reported positive correlations between the abundances of *Blautia*, *Bifidobacterium*, and *Eisenbergiella* and skeletal MM. Interestingly, another study showed that elevated *Blautia* levels were paradoxically linked to lower skeletal MM in older Japanese adults [36], suggesting strain- or context-specific effects. A systematic review and fecal metagenomic analysis further reported a consistent decrease in SCFA-producing bacteria and associated functional genes involved in SCFA synthesis, carotenoid and isoflavone biotransformation, and amino acid interconversion in SA individuals [37,38].

In line with previous findings, our results confirm similar microbial alterations in a Japanese clinical cohort using AWGS 2019 diagnostic criteria. In particular, *Fusicatenibacter* was decreased in the SA group and positively correlated with grip strength and gait speed. Previous research linked *Fusicatenibacter* to anaerobic performance and stool characteristics in Japanese athletes [39]. Taxonomic studies have shown that this genus produces SCFAs, which are absorbed through the colon and utilized in liver and muscle tissues as substrates for glycogen and fatty acid synthesis [40,41]. Nay et al. demonstrated that depletion of GM by antibiotics in mice led to reduced endurance and impaired muscle function, potentially because of muscle glycogen depletion [42].

Similarly, *Enterococcus H* was positively correlated with grip strength and gait speed, suggesting its potential involvement in host muscle metabolic pathways. Liu et al. [43] demonstrated that butyrate supplementation in SA mice restored MM and function via mTOR pathway activation. Mayer et al. [44] reported that lower diversity of butyrate-producing bacteria was related to decreased grip strength and physical performance in older adults. Collectively, these results reinforce the importance of SCFA-producing bacteria in muscle maintenance through nutrient-sensing and anabolic pathways.

*Eubacterium I*, the genus most strongly associated with SA in our analysis, may correspond to *E. ramulus* based on 16S rRNA BLAST (ver. BLAST+ 2.15.0) results. *E. ramulus* can metabolize dietary flavonoids, and its abundance increases following polyphenol intake [45,46]. It expresses key enzymes, such as chalcone isomerase and NADH-dependent reductases, involved in flavonoid biotransformation [47,48,49]. According to a review on the potential of flavonoids for muscle atrophy, multiple in vitro and in vivo studies have suggested that flavonoids are potential therapeutics for treating muscle atrophy [50,51]. Moreover, dietary diversity, including vegetable and fruit intake—rich sources of polyphenols—has been associated with better muscle health and reduced SA risk [52,53]. Our previous findings also support that low vegetable intake correlates with SA in patients with chronic kidney disease [54,55], possibly via reduced availability of bacteria such as *E. ramulus*, which metabolize beneficial compounds. Thus, microbiota-mediated polyphenol metabolism may represent an underexplored pathway in muscle preservation. Several mechanisms have been proposed through which short-chain fatty acids (SCFAs) may influence SA. First, SCFAs possess anti-inflammatory properties that may indirectly support muscle protein synthesis and maintenance by suppressing chronic inflammation [9]. In particular, butyrate has been shown to activate key signaling pathways such as MAPK and mTOR in C2C12 myoblasts, potentially promoting muscle anabolism [56]. Additionally, SCFAs have been reported to improve insulin sensitivity, which may further contribute to the enhancement of muscle protein synthesis and preservation [8].

Recent studies have shown that targeted interventions such as probiotic supplementation can mitigate these effects. For instance, *Lactobacillus plantarum* TWK10 has been demonstrated to improve muscle mass and strength in older adults. *Bifidobacterium longum* and *Lactobacillus casei* strains have been associated with reduced inflammation and improved muscle function in animal models [57,58]. Further investigation of the effect of probiotic administration on the symptoms of SA in a large RCT trial is warranted.

This study had several limitations. First, the sample consisted of outpatients at a single center, potentially limiting the generalizability of our findings. Second, potential confounding factors such as exercise routines, dietary patterns, occupation, education level, and marital status were not accounted for. Considering that a range of daily lifestyle behaviors has been shown to influence the risk of SA among older adults, incorporating these factors into clinical assessments and preventive strategies is essential [59]. Third, the cross-sectional design precludes causal inference between GM and SA; therefore, prospective intervention studies are warranted. Fourth, in this study, the different proportions of male and female patients in the subject. Finally, it is important to acknowledge the limitations of 16S rRNA sequencing, which offers only genus-level taxonomic resolution. To gain a more comprehensive understanding of the role of the GM in SA, the application of more advanced approaches—such as whole metagenomic and metabolomic analyses—should be emphasized. This study was conducted as a single-center cross-sectional analysis and therefore may not represent data reflective of the entire Japanese population. To address regional variability and ensure broader applicability, future research should include multicenter collaborative studies and prospective investigations directly comparing Japanese cohorts with Western populations.

## 5. Conclusions

This prospective cross-sectional study revealed a potential association between GM composition and SA, as defined by the AWGS 2019 criteria, in elderly outpatients in Japan. Notably, the relationship between SA and GM composition appeared to differ by sex. In males, the SA group exhibited lower alpha diversity and significant differences in beta diversity compared to the non-SA group. Additionally, six bacterial genera—*Eubacterium I*, *Fusicatenibacter*, *Holdemanella*, *Unclassified Lachnospira*, *Enterococcus H*, and *Bariatricus*—were less abundant in the SA group, and four of these exhibited lower detection rates. Several of these genera produce SCFA, particularly butyrate, and were positively correlated with MM, grip strength, and gait speed. These findings suggest that alterations in GM composition, particularly reductions in SCFA-producing bacteria, may be linked to SA in elderly males. Microbiota-targeted interventions, including dietary modifications and probiotic supplementation, could represent promising strategies for SA prevention and treatment. However, the cross-sectional study design limits causal inference, and further longitudinal and interventional studies are necessary to confirm these associations and clarify the underlying mechanisms.

## Figures and Tables

**Table 1:** Background information of participants with and without sarcopenia.

	All Subjects Without Sarcopenia (*n* = 306)	All Subjects with Sarcopenia (*n* = 50)	*p*-Values	Male Subjects Without Sarcopenia (*n* = 109)	Male Subjects with Sarcopenia (*n* = 35)	*p*-Values	Female Subjects Without Sarcopenia (*n* = 197)	Female Subjects with Sarcopenia (*n* = 15)	*p*-Values
Age	76.9 ± 6.1	79.5 ± 6.9	0.011 *	76.1 ± 5.8	79.4 ± 6.7	0.004 **	77.0 ± 6.2	78.9 ± 6.4	0.530
Hight (m)	1.56 ± 0.10	1.58 ± 0.09	0.242	1.67 ± 0.06	1.62 ± 0.07	0.002 **	1.50 ± 0.06	1.51 ± 0.04	0.794
Body weight (kg)	57.0 ± 11.9	52.8 ± 9.3	0.057	67.3 ± 9.9	56.8 ± 7.4	0.000 **	51.2 ± 8.5	42.9 ± 4.8	0.003 **
BMI	23.3 ± 3.7	21.2 ± 2.7	0.000 **	24.3 ± 3.4	21.7 ± 2.6	0.001 **	22.8 ± 3.8	19.0 ± 2.4	0.009 **
SMI (kg/m^2^)	6.37 ± 0.97	5.80 ± 0.81	0.000 **	6.99 ± 0.90	6.15 ± 0.63	0.000 **	6.07 ± 0.84	5.31 ± 0.65	0.000 **
Grip strength (kg)	26.5 ± 9.0	24.9 ± 8.3	0.383	34.8 ± 8.2	27.9 ± 7.1	0.000 **	22.3 ± 5.7	19.0 ± 6.7	0.000 **
Walking speed (m/s)	1.28 ± 0.41	0.96 ± 0.31	0.000 **	1.41 ± 0.44	1.00 ± 0.28	0.000 **	1.23 ± 0.38	0.94 ± 0.25	0.005 **

* *p* < 0.05, ** *p* < 0.01.

**Table 2:** Detection rates of bacterial genera in sarcopenic and non-sarcopenic patients.

Detection Ratio	Male Non-Sarcopenia	Male Sarcopenia	Fisher’s Exact Test	Female Non-Sarcopenia	Female Sarcopenia	Fisher’s Exact Test
p__Firmicutes_A|c__Clostridia_258483|o__Lachnospirales|f__Lachnospiraceae|g__Eubacterium_I	61%	23%	0.0002 **	37%	27%	0.581
p__Firmicutes_A|c__Clostridia_258483|o__Lachnospirales|f__Lachnospiraceae|g__Fusicatenibacter	100%	97%	0.243	97%	100%	1.000
p__Firmicutes_D|c__Bacilli|o__Erysipelotrichales|f__Erysipelotrichaceae|g__Holdemanella	71%	46%	0.009 **	46%	33%	0.427
p__Firmicutes_D|c__Bacilli|o__Lactobacillales|f__Enterococcaceae|g__Enterococcus_H_360604	42%	14%	0.002 **	28%	40%	0.376
p__Firmicutes_A|c__Clostridia_258483|o__Lachnospirales|f__Lachnospiraceae|__	98%	91%	0.093	91%	100%	0.622
p__Firmicutes_A|c__Clostridia_258483|o__Lachnospirales|f__Lachnospiraceae|g__Bariatricus	49%	23%	0.010 *	32%	20%	0.401

* *p* < 0.05, ** *p* < 0.01.

## Data Availability

Data supporting the findings of this study are available upon request from the corresponding author. The data is not shared publicly due to being a part of an ongoing study.

## Supplementary Materials

The following supporting information can be downloaded at https://www.mdpi.com/article/10.3390/nu17101746/s1, Figure S1: Principal component analysis based on Bray Curtis (β-diversity) analysis between male and female participants.

## Abbreviations

The following abbreviations are used in this manuscript:

GM	gut microbiota
SCFA	short-chain fatty acid
AWGS	Asian Working Group for Sarcopenia
MM	muscle mass
MS	muscle strength
SMI	skeletal muscle mass index
SA	sarcopenia
