# Supplementary material for: Sex-Specific Associations of Gut Microbiota Composition with Sarcopenia Defined by the Asian Working Group for Sarcopenia 2019 Consensus in Older Outpatients: Prospective Cross-Sectional Study in Japan
Source: Nutrients. 2025 May 21;17(10):1746. doi: 10.3390/nu17101746 (PMC12114429; doi:10.3390/nu17101746)
Supplement: Supplementary file 1 [file nutrients-17-01746-s001.zip › nutrients-3653454-supplementary.pdf]

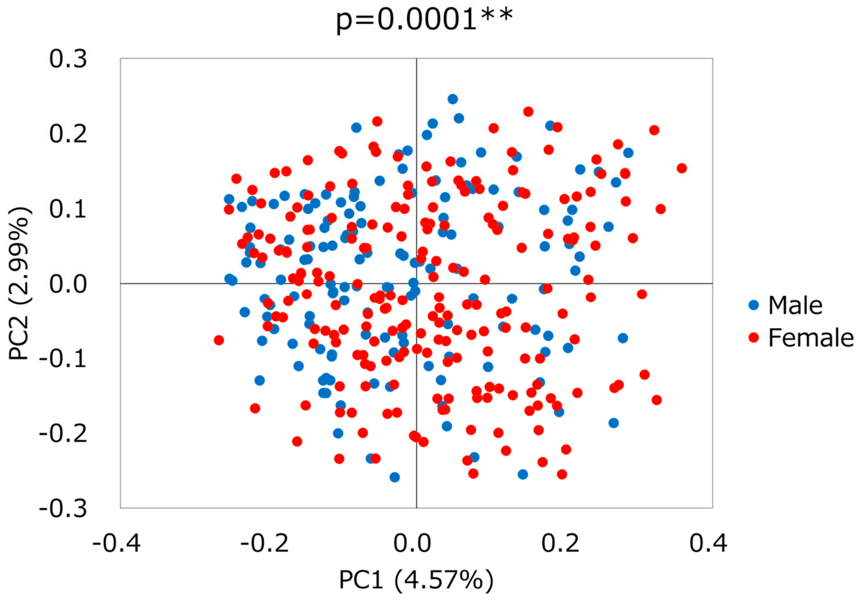

Supplementary Figure S1. Principal component analysis based on Bray Curtis (β-diversity) analysis between male and female participants

PCoA was used to visualize the differences in gut microbiota comparing male and female. PCoA plots show the beta-diversity with Bray–Curtis dissimilarity. Each dot represents an individual participant. The statistical significance of differences in beta-diversity was analysed using PERMANOVA (**$p<0.01$).
